# Impaired response of blood neutrophils to cell-death stimulus differentiates AQP4-IgG-seropositive NMOSD from MOGAD

**DOI:** 10.1186/s12974-022-02600-0

**Published:** 2022-10-01

**Authors:** Maria Schroeder-Castagno, Alba Del Rio-Serrato, Andreas Wilhelm, Silvina Romero-Suárez, Patrick Schindler, Cesar Alvarez-González, Ankelien-Solveig Duchow, Judith Bellmann-Strobl, Klemens Ruprecht, Maria Hastermann, Gerald Grütz, Brigitte Wildemann, Sven Jarius, Tanja Schmitz-Hübsch, Friedemann Paul, Carmen Infante-Duarte

**Affiliations:** 1 Charité-Universitätsmedizin Berlin, Corporate Member of Freie Universität Berlin and Humboldt-Universität Zu Berlin, ECRC Experimental and Clinical Research Center, a Cooperation Between the Max Delbrück Center for Molecular Medicine in the Helmholtz Association and Charité-Universitätsmedizin Berlin, Lindenberger Weg 80, 13125 Berlin, Germany; 2 Max Delbrück Center for Molecular Medicine in the Helmholtz Association (MDC), Campus Berlin-Buch GmbH, Robert-Rössle-Straße 10, 13125 Berlin, Germany; 3 Institute for Medical Immunology, Charité-Universitätsmedizin Berlin, Campus Virchow Klinikum, Augustenburger Platz 1, 13353 Berlin, Germany; 4 BIH Center for Regenerative Therapies (BCRT) Charité- Humboldt-Universität Zu Berlin and Berlin Institute of Health, Institute for Medical Immunology, Core Unit Immunocheck-Biomarker Immunologisches Studienlabor (ISL), Campus Virchow Klinikum, Augustenburger Platz 1, 13353 Berlin, Germany; 5 Charité-Universitätsmedizin Berlin, Corporate Member of Freie Universität Berlin, Humboldt-Universität Zu Berlin, NeuroCure Clinical Research Center, Campus Mitte, Charitéplatz 1, 10117 Berlin, Germany; 6 Department of Neurology, Charité-Universitätsmedizin Berlin, Campus Mitte, Charitéplatz 1, 10117 Berlin, Germany; 7 Molecular Neuroimmunology Group, Department of Neurology, University of Heidelberg, Heidelberg, Germany; 8 Department of Immunobiochemistry, Mannheim Institute for Innate Immunoscience (MI3), Medical Faculty Mannheim, University of Heidelberg, 68167 Mannheim, Germany; 9 Neurologic Clinic and Policlinic, Departments of Medicine, University Hospital Basel & RC2NB - Research Center for Clinical Neuroimmunology and Neuroscience, University of Basel, Basel, Switzerland

**Keywords:** Aquaporin-4 NMOSD, Neuromyelitis optica spectrum disorders, MOGAD, Myelin oligodendrocyte glycoprotein-antibody-associated disease, Neutrophils

## Abstract

**Background:**

In neuromyelitis optica spectrum disorders (NMOSD) and myelin oligodendrocyte glycoprotein antibody-associated disease (MOGAD), neutrophils are found in CNS lesions. We previously demonstrated that NMOSD neutrophils show functional deficiencies. Thus, we hypothesized that neutrophil accumulation in the CNS may be facilitated by impairments affecting mechanisms of neutrophil death.

**Objective:**

To evaluate cell death in blood neutrophils from aquaporin-4 (AQP4)-IgG-seropositive NMOSD and MOGAD patients as well as matched healthy controls (HC) using in vitro assays.

**Methods:**

Twenty-eight AQP4 + NMOSD and 19 MOGAD patients in stable disease phase as well as 45 age- and sex-matched HC were prospectively recruited. To induce cell death, isolated neutrophils were cultured with/without phorbol 12-myristate 13-acetate (PMA). Spontaneous and PMA-induced NETosis and apoptosis were analyzed using 7-AAD and annexin-V by flow cytometry. Caspase-3 was assessed by western blot. Myeloperoxidase-DNA complexes (MPO-DNA), MPO and elastase were evaluated by ELISA, and cell-free DNA (cfDNA) by a fluorescence-based assay. Reactive oxygen species (ROS) were evaluated by a dihydrorhodamine 123-based cytometric assay. Serum GM-CSF, IL-6, IL-8, IL-15, TNF-ɑ and IL-10 were evaluated by multiplex assays, and neurofilament light chain (NfL) by single-molecule array assay.

**Results:**

In response to PMA, neutrophils from AQP4 + NMOSD but not from MOGAD patients showed an increased survival, and subsequent reduced cell death (29.6% annexin V^+^ 7-AAD^+^) when compared to HC (44.7%, *p* = 0.0006). However, AQP4 + NMOSD also showed a mild increase in annexin V^+^ 7-AAD^−^ early apoptotic neutrophils (24.5%) compared to HC (20.8%, *p* = 0.048). PMA-induced reduction of caspase-3 activation was more pronounced in HC (*p* = 0.020) than in AQP4 + NMOSD neutrophils (*p* = 0.052). No differences were observed in neutrophil-derived MPO-DNA or serum levels of MPO, elastase, IL-6, IL-8 and TNF-ɑ. IL-15 levels were increased in both groups of patients. In AQP4 + NMOSD, an increase in cfDNA, GM-CSF and IL-10 was found in serum. A positive correlation among cfDNA and NfL was found in AQP4 + NMOSD.

**Conclusions:**

AQP4 + NMOSD neutrophils showed an increased survival capacity in response to PMA when compared to matched HC neutrophils. Although the data indicate that the apoptotic but not the NETotic response is altered in these neutrophils, additional evaluations are required to validate this observation.

**Supplementary Information:**

The online version contains supplementary material available at 10.1186/s12974-022-02600-0.

## Background

Neuromyelitis optica spectrum disorders (NMOSD) encompass a group of severe antibody-mediated autoimmune diseases of the central nervous system (CNS), long believed to be rare variants of multiple sclerosis (MS) [1, 2]. Serum auto-antibodies against aquaporin-4 (AQP4-IgG) are found in ≥ 80% of NMOSD patients [2, 3]. Anti-AQP4-IgG (herein referred to as AQP4 +) have been shown to cause an astrocytopathy that is accompanied by infiltration of granulocytes and macrophages into the CNS, and to lead to a secondary loss of oligodendrocytes and neurons [2]. However, the pathophysiology of NMOSD remains incompletely understood, especially in the group of patients that are seronegative for anti-AQP4-IgG. About 10–40% of those seronegative patients are seropositive for autoantibodies against myelin oligodendrocyte glycoprotein (MOG-IgG) [2]. Although AQP4 + NMOSD, as defined by the International Panel for Neuromyelitis Optica (NMO) Diagnosis (IPND) criteria [4], and MOG-IgG-seropositive patients present comparable clinical and radiological features, current data support the assumption that the presence of anti-MOG-IgG defines a disorder pathogenetically distinct from both NMOSD and MS [5–9], and which has been recently termed myelin oligodendrocyte glycoprotein (MOG)-associated encephalomyelitis (MOG-EM) [5] or MOG antibody-associated disease (MOGAD) [10].

Perivascular granulocytes, principally neutrophils and eosinophils, are found in active lesions of patients during the early phase of NMOSD [11–13], and in the cerebrospinal fluid (CSF) during relapses and remission [14]. An increase in the circulating neutrophil-to-lymphocyte ratio (NLR) has been reported during relapse in both, APQ4 + NMOSD and MOGAD [15, 16], but during remission only in APQ4 + NMOSD patients compared to healthy controls (HC) [16]. Neutrophils are also found in the CNS lesions of NMO animal models [17–21]. Moreover, increasing evidence suggests a contribution of neutrophils to the deleterious inflammatory cascade inside the CNS [11, 17–27]. In line with these findings, our previous study showed that both NMOSD and MS-derived neutrophils have an activated phenotype in comparison to HC. However, compared to MS, NMOSD display deficient functionalities such as reduced N-formyl-L-methionyl-L-leucyl-phenylalanine (fMLP)-induced neutrophil migration and oxidative burst [25]. We therefore hypothesized that neutrophils from AQP4 + NMOSD and MOGAD patients might have an impaired response to cell-death stimuli, supporting their pathological accumulation in CNS lesions. Neutrophil death and clearance are crucial for resolution of inflammation and for preventing tissue damage. Neutrophils can undergo different death programs, including apoptosis or NETosis, a programmed cell death that involves the formation and release of DNA-based neutrophil extracellular traps [28, 29]. Thus, in the present study we evaluated the susceptibility of neutrophils from AQP4 + NMOSD and MOGAD patients to undergo cell death using in vitro assays and investigated the altered cell-death mechanisms.

## Material and methods

### Study participants

Patients as well as age- and sex-matched healthy controls (HC) were recruited at the NeuroCure Clinical Research Center, the Experimental and Clinical Research Center and the Institute for Medical Immunology, Charité-Universitätsmedizin Berlin, between February 2017 and August 2021, and participated in an ongoing prospective study of patients with NMOSD and related disorders. The study was approved by the Ethics Committee of the Charité-Universitätsmedizin Berlin (EA1/041/14, EA1/163/12) and conducted in accordance with the Helsinski declaration. Written informed consent was obtained from all participants. Inclusion criteria for patients included: clinical remission for at least 2 months before blood collection, age ≥ 18 years old, diagnosis of AQP4 + NMOSD according to the 2015 IPND consensus criteria [4], or diagnosis of MOGAD according to the following criteria: at least one clinical characteristic (optic neuritis, acute myelitis), AQP4-IgG seronegative and seropositive for MOG-IgG in a cell-based assay at least once during their disease course and exclusion of alternative diagnoses, according to the criteria of Jarius and colleagues [5]. Patients with a concomitant autoimmune disease (i.e., systemic lupus erythematosus (SLE), Sjögren syndrome; see Table 1) were not excluded from the study as this frequently occurs in NMOSD [30]. At the study visit, all patients were examined neurologically and scored applying the Kurtzke Expanded Disability Status Scale 10 (EDSS) [31]. The annualized relapse rate (ARR) was calculated considering the number of relapses experienced by the patients from disease onset (see Table 1) as previously described [32].

**Table 1:** Epidemiological and clinical characteristics of study population

	HC	AQP4 + NMOSD	MOGAD
Number, *n*	45	28	19
Age, years, mean (SD)	46 (13)	51 (13)	46 (16)
Female/male, *n/n* (% female)	38/7 (84)	27/1 (97)	13/6 (68)
*Serostatus*, *n/n*			
AQP4-IgG	n.a	28/28	0/19
MOG-IgG	n.a	0/28	19/19
Time from disease onset to visit, years, median (IQR)	n.a	8.4 (2.7–9.8)	4.5 (1.7–7)
ARR median (range) Number of patients with at least 1 attack during previous year, *n* (%)	n.a n.a	0.39 (0.12–4.94) 5 (18)	0.73 (0.12–3.38) 7 (37)
*Type of last attack*, *n* (%)			
Optic neuritis	n.a	2 (7)	4 (21)
Myelitis Brainstem encephalitis	n.a n.a	3 (11) 0 (0)	2 (11) 1 (6)
EDSS, median (IQR)	n.a	3(2–4)	2 (1.5–3.5)
Immunotherapy, *n/n* (%)	No:45/45 (100)	No:6/28 (21) Any:22/28 (79) RTX:14/28 (50) AZA:6/28 (21) MMF:2/28 (7) TCZ:1/28 (3) ECU:1/28 (3)	No:5/19 (26) Any:14/19 (74) RTX:8/19 (42) AZA:1/19 (10) MMF:2/19 (10) TCZ:1/19 (5) MTX:1/19 (5) IVIG:1/19 (5)
*Concomitant autoimmune disease*, *n/n* (%)	n.a	7/28 (25)	1/19 (5)
SLE	n.a	4/28 (14)	0/19 (0)
Sjögren syndrome	n.a	2/28 (7)	0/19 (0)
Myasthenia gravis	n.a	2/28 (7)	0/19 (0)
Autoimmune thyroiditis	n.a	2/28 (7)	1/19 (5)

*HC* healthy control*, AQP4* + anti-aquaporin-4 IgG-seropositive*, NMOSD* neuromyelitis optica spectrum disorders**,** *MOGAD* myelin oligodendrocyte glycoprotein-antibody-associated disease, *n* number, *SD* standard deviation, *IQR* interquartile range, *EDSS* Expanded Disability Status Scale, *n.a.* not applicable; *RTX* rituximab; *AZA* azathioprine; *MMF* mycophenolate mofetil; *TCZ* tocilizumab; *ECU* eculizumab; *MTX* methotrexate; *IVIG* intravenous immunoglobulin, *No:* not treated with RTX; AZA, MMF, TCZ, ECU, MTX, steroids, IVIG or had plasma exchange for 2 months prior to sample collection, *SLE* systemic lupus erythematosus. EDSS missing data for AQP4 + NMOSD *n* = *1* in visit date (not shown)

Untreated patients did not receive immunosuppressive medication such as azathioprine, rituximab, mycophenolate mofetil, methotrexate, mitoxantrone, steroids or intravenous immunoglobulin (IVIG), or were not subjected to plasma exchange for at least two months before blood collection.

### Standard protocol approvals, registrations, and patient consents

This study was evaluated and approved by the Ethics Committee of the Charité-Universitätsmedizin Berlin (EA1/041/14 and EA1/163/12). The participants provided their written informed consent to participate in this study.

### Antibody detection in serum

Serum anti-AQP4-IgG and anti-MOG-IgG was tested using cell-based assays based on HEK293 cells that are transfected with either full-length human AQP4 or full-length human MOG (Molecular Neuroimmunology Group, University of Heidelberg, Germany) [33, 34]. Patients were classified as seropositive for anti-AQP4-IgG or anti-MOG-IgG if they tested positive for one of these antibodies at least once during their disease course.

### Sample collection

Due to their short lifespan and neutrophils’ marked sensitivity to environmental conditions, fresh, heparinized peripheral blood samples from patients and their corresponding HC were collected within 45 min of venipuncture and processed simultaneously using identical protocols. Patient and HC sera were obtained, processed, and stored following standard operating procedures (SOP). Whole blood from patients and HC was used for ROS analysis as described below.

### Neutrophil isolation and in vitro induction of neutrophil death with PMA

Samples from AQP4 + NMOSD and MOGAD patients and their age- and sex-matched HC were collected and processed simultaneously. Neutrophil isolation from peripheral venous blood of HC and patients was done following the protocol of Clark and Nauseef [35]. In brief, neutrophils were separated from erythrocytes and mononuclear cells by sedimentation on a separating medium of 3% dextran (Sigma-Aldrich, Germany) in Hanks’ buffered salt solution (Gibco Life Technologies, Germany), followed by Ficoll centrifugation (Biochrom-Merck, Germany). Erythrocytes were removed by using hypotonic lysis buffer (Biolegend, Germany). Percent viability of neutrophils was determined by trypan blue dye staining (ThermoFisher Scientific, Germany) and showed a purity > 95%. Afterwards, cells were resuspended at 2 × 10^6^ cells/ml in RPMI 1640 (Gibco Life Technologies, Germany) supplemented with 10% heat-inactivated fetal bovine serum (Biochrom-Merck, Germany) and 1% penicillin/streptomycin (ThermoScientific, Germany). To induce cell death, neutrophil suspension was seeded into 96-well plates and incubated with 25 nM of phorbol 12-myristate 13-acetate (PMA, Sigma-Aldrich, Germany) for 30 min at 37 °C in a humidified atmosphere of 5% CO2 and 95% air at 37 °C. Neutrophils incubated only with vehicle dimethyl sulfoxide (DMSO) were used as controls and are shown as “*none”*. After incubation with PMA or vehicle, cells were washed and resuspended in phosphate-buffered saline (PBS) for posterior cell death evaluation by flow cytometry 2.5 h after PMA stimulation initiation or cell lysates, and culture supernatants were collected, harvested 2.5 h after PMA stimulation initiation.

### Ex vivo analysis of neutrophil death by flow cytometry

Spontaneous and PMA-induced cell death was evaluated by flow cytometry at 2.5 h after starting the PMA stimulation in samples from patients and their age and sex-matched HC collected and processed simultaneously with identical protocols. To assess apoptosis and NETosis, neutrophils were stained with 7-amino-actinomycin D (7-AAD; nuclear staining) and annexin V (phosphatidylserine staining) according to manufacturer’s instructions (Biolegend, Germany) after a previous incubation period of 15 min with an Fc receptor-blocking antibody (1:20; Miltenyi Biotec, Germany) and staining with anti-human CD16b-Pacific Blue antibody (1:500, clone MEM-154; Antikörper Online, Germany) as previously described [25]. Subsequently, living and dying neutrophils were investigated by flow cytometry (BD LSRFortessa; BD Biosciences, USA), and the data were analyzed using FlowJo (v10.4; BD, USA). Annexin V^−^ 7-AAD^−^ were considered living neutrophils; early apoptotic neutrophils were defined as annexin V^+^ 7-AAD^−^. Annexin V^+^ 7-AAD^+^ cells included dying neutrophils in late apoptosis or NETosis. Neutrophils were identified by their size (FSC-A) and granularity (SSC-A) and by the expression of CD16b. Neutrophil purity was ≥ 92% as assessed by expression of a neutrophil-specific marker (CD16b).

### Protein extraction and western blot analysis

Samples from AQP4 + NMOSD, MOGAD patients and age- and sex-matched HC were collected. Neutrophil lysates were generated from neutrophils seeded at 2 × 10^6^ cells/ml and incubated with/without (*none*) 25 nM of PMA at 37 °C in a humidified atmosphere of 5% CO_2_ and 95% air. Cell lysis was performed in RIPA buffer (50 mM Tris–HCl pH 8, 150 mM NaCl, 5 mM EDTA, 1% Triton-X 100, 0.25% sodium deoxycholate, 0.1% SDS) and supplemented with a protease inhibitor cocktail (Sigma-Aldrich, Germany) for 45 min on ice. Thereafter, neutrophils cell lysates were pelleted and quantified by bicinchoninic acid assay (ThermoScientific, USA) according to the manufacturer’s instructions. Afterwards, cell lysates were dissolved in protein loading buffer (LI-COR, USA) with 10% β-mercapto-ethanol, heated for 5 min at 95 °C, cooled at room temperature (RT) and then stored at -20 °C until use. For analysis, equal amount of total protein was loaded and run on 12.5% SDS-PAGE (polyacrylamide gel electrophoresis) in a Bio Rad Chamber and subsequently transferred to polyvinyl-difluoride membranes (PDVF; Merck Millipore, Germany). Membranes were blocked for 1.5 h at RT with Odyssey blocking buffer (LI-COR, USA) and then washed in Tris-buffered saline, 0.1% Tween 20 (TBST).

Blots were immuno-labeled with rabbit primary antibodies directed against inactive caspase-3 (full length; Pro-caspase-3) and cleaved caspase-3 fragments, respectively (1:1000; Cell Signaling, Germany) together with rabbit anti-human β-tubulin (1:1000; Cell Signaling, Germany) as a loading control protein. Incubation was done overnight at 4 °C in TBS-T-5% dry milk. Subsequently, membranes were washed and incubated with a secondary donkey anti-rabbit-IgG conjugated to infra-red dye (1:15,000; LI-COR, USA) in TBS-T-0.1% dry milk for 1 h at RT. Inactive caspase-3 (Pro-caspase-3), active caspase-3 and β-tubulin proteins were visualized using the Odyssey near-infrared imaging system (LICOR, USA). Band fluorescence intensities were quantified using Image Studio densitometry software (v.5.2; LI-COR, USA). The intensity units of bands were calculated by the subtraction of the background signal. Caspase-3 signal intensity values (17 and 19kDa bands were analyzed together), were normalized in relation to the values of the control β-tubulin and are shown as caspase-3 arbitrary units (A.U.).

### Ex vivo assessment of granulocyte ROS production by flow cytometry

Whole blood from AQP4 + NMOSD and MOGAD patients as well as age- and sex-matched HC was used to assess the production of ROS by granulocytes upon PMA stimulation. Unstimulated samples were used as controls. ROS production was evaluated by Labor Berlin, following SOPs according to the manufacturer’s instructions (Phagoburst test; CELONIC, Germany). In brief, samples were stimulated with PMA and incubated with fluorogenic substrate dihydrorhodamine 123 (DHR 123). Intracellular ROS were indirectly assessed by flow cytometry (Navios EX-flow Cytometer; Beckman Coulter, USA). DHR 123 is a nonfluorescent molecule that diffuses across cell membranes and is oxidized by hydrogen peroxide (H2O2) resulting in the formation of a fluorescent dye (oxidized-DHR 123). Granulocytes were identified by their characteristic size and granularity in the sideward and forward scatter graph. Mean fluorescence intensity of oxidized-DHR 123 was calculated using Navios EX-flow Cytometer software.

### Immunocytochemical staining and neutrophil cell death evaluation by fluorescence microscopy

Neutrophils obtained from AQP4 + NMOSD, MOGAD and HC were processed simultaneously, applying the same isolation and staining protocols as previously described [28]. In brief, isolated neutrophils were seeded at 2 × 10^5^ cells/ml per well over poly-L-lysine coated 12-mm round glass coverslips (Corning, BD, Biosciences, USA). Cells were allowed to settle for 30 min at 37 ºC. Afterwards, neutrophils were stimulated with 25 nM PMA or left unstimulated (*none*) for a period of 2.5 h. Subsequently, cells were fixed with 2% paraformaldehyde (PFA, Electron Microscopy Sciences, England) and then stored overnight at 4 ºC. On the following day, coverslips were removed from the plate and washed with floating drops of PBS on hydrophobic laboratory film and then permeabilized with 0.5% Triton-X 100 in PBS (ThermoScientific, Germany) for 5 min at RT. Afterwards, coverslips were washed and samples blocked for 30 min at RT in blocking buffer containing 3% donkey serum (Sigma-Aldrich, Germany), 3% cold-water fish gelatin (Biotium, USA), 1% bovine serum albumin (Miltenyi Biotec, Germany), and 0.05% Tween 20 (ThermoScientific, Germany) in PBS. For immunostaining, coverslips were incubated with primary rabbit anti-human elastase antibody (1:500, Calbiochem, Germany) in blocking buffer for 2 h at RT. After washing, coverslips were incubated with the secondary goat anti-rabbit Alexa Fluor-488 antibody (1:500, Invitrogen, Germany) for 1 h at RT. For nuclear staining, samples were incubated with aqueous Hoechst 33342 (1:1500, Immunochemistry Technologies, USA) in blocking buffer for 30 min at RT, washed and then mounted in Prolong Diamond antifade mounting solution (ThermoScientific, Germany). Negative staining control coverslips for each sample were prepared following the same protocol, without primary antibody addition. Images of a minimum of eight fields of each coverslip were acquired using an inverted fluorescence phase contrast microscope BZ-X800/BZ-X810 with a 40× objective. Images were then loaded and analyzed in a blinded manner with CellProfiler 4.2.1 software. Nuclei were identified and quantified based on Hoechst signal and size. NETosis values were determined by quantification of NET-like structures with expanded nuclei or extracellular DNA in elastase-positive neutrophils, co-localized with Hoechst signal. NETosis area was calculated as the mean value of the area occupied by elastase, in at least eight fields (40x), normalized to the total number of cells and shown as NETosis (A.U.). One AQP4 + NMOSD *(none)* and one matched HC sample *(none)* could not be analyzed due to technical problems. To assess the formation of DNA fragments during neutrophils late apoptosis, terminal deoxynucleotidyl transferase-nick end labeling (TUNEL) staining was performed in coverslips according to kit manufacturer’s instructions (In Situ Cell Death Detection Kit, TMR Red, Roche Diagnostics, Germany).

### Evaluation of soluble markers of cell death in neutrophil culture supernatants and serum samples

Cell-free double strand DNA (cfDNA), myeloperoxidase–dsDNA complexes (MPO-DNA), MPO and neutrophil elastase were investigated in culture supernatants and serum samples from AQP4 + NMOSD and MOGAD patients as well as age- and sex-matched HC. CfDNA was analyzed using the Quant-iT Picogreen DNA kit (ThermoScientific, Germany) in accordance with manufacturer’s instructions. MPO-DNA complexes were identified using a capture ELISA as described previously [36]. In brief, 5 µg/ml of mouse anti-human MPO monoclonal antibody (Abcam, Germany) was coated to Nunc™ Maxisorp™ 96-well high bind microplate (Abcam, Germany) overnight at 4 ºC in coating buffer (Abcam, Germany). Plates were washed, and blocking buffer (Abcam, Germany) was added for 2 h at RT. After washing, samples were added in combination with peroxidase-labeled anti-DNA monoclonal antibody (component 2 Cell Death ELISA kit: Roche, Germany). After 2 h of incubation at RT on a shaking device, samples were washed. For MPO-DNA complexes detection, peroxidase substrate (3.3,5,5-tetramethylbenzidine) was added. After 15 min of incubation at RT in the dark, stop solution (1 M H3PO4) was added, and the optical absorbance at 450 nm was measured using the GloMax plate reader (Promega, Germany). For the evaluation of MPO-DNA complexes in culture supernatant, neutrophils were treated as described above, either without (*none*) or with 25 nM of PMA stimulation for 2.5 h. Culture supernatants were subsequently harvested in both PMA-stimulated and *none* groups, and stored at − 80 °C until analysis. Levels of MPO-DNA were analyzed in all samples in parallel. Before measurement, neutrophil culture supernatants were diluted 1:8 in double-distilled water (Gibco Life Technologies, Germany). Myeloperoxidase (MPO) and elastase serum levels were determined by ELISA kit (Abcam, Germany), according to manufacturer’s instructions.

### Evaluation of cytokines in serum samples of AQP4 + NMOSD and MOGAD patients and HC

Levels of GM-CSF, IL-6, IL-8, IL-15, TNF-ɑ and IL-10 were investigated at the Immunodiagnostic Core Unit of the BIH Center for Regenerative Therapies—Immunocheck Lab at Charité—Universitätsmedizin Berlin. The Meso Scale Diagnostics MULTISPOT Assay System (human proinflammatory panel 1 kit for IL-10, IL-6, IL-8 and TNF-α, and cytokine panel 1 for GM-CSF and IL-15) was used according to SOPs. Serum samples from AQP4 + NMOSD and MOGAD patients and their age- and sex-matched HC were collected and processed simultaneously. The optical density values of each analyte were calculated using a calibration curve and after blank subtraction. The concentration of each serum analyte was calculated using its respective standard curve. The detection limits of the assays, low detection limit; high limit of detection (LLOD-HLOD) for the different cytokines were GM-CSF (0.140–808 pg/ml), IL-6 (0.086-684 pg/ml), IL-8 (0.023–633), IL-15 (0.115–976 pg/ml), TNF-ɑ (0.034–371 pg/ml), and IL-10 (0.012–334 pg/ml).

### Evaluation of neurofilament light chain in serum

Serum samples were collected and centrifuged at 2000*g* for 10 min at RT and then stored at − 80 °C until analysis. For neurofilament light chain (NfL) measurement, we employed single-molecule array immunoassay technology (Simoa) as used in other studies [37, 38].

### Data analysis

Data were analyzed using FlowJo (v10.4; BD, USA), Navios EX-flow Cytometer (Beckman Coulter, USA), GraphPad Prism 8 (v.8.0.1; GraphPad, USA), Image Studio (v.5.2; LI-COR, USA) and CellProfiler 4.2.1 softwares. Graphs were drawn, and statistical data analyses were performed, with GraphPad Prism 8. Continuous variables are expressed as median and interquartile range (IQR) or mean ± SD or SEM, indicated in figure legends. Nonparametric statistical methods were used. Comparisons among two paired groups, patients and their age- and sex-matched HC, or comparison of the same subject with and without PMA stimulus, were done with non-parametric Wilcoxon signed-rank test. Mann–Whitney U test was used for non-matched group comparisons (Fig. 4). A two-tailed *p*-value less (˂) than 0.05 was considered statistically significant; *p*-values > 0.05 and < 0.07 were considered as statistic trends and are also shown in figures. Significant statistical differences are shown with (*), while *p*-values < 0.01 are shown with (**), *p*-values ˂0.001 with (***), and *p*-values ˂0.0001 with (****). Due to the exploratory nature of the study, no adjustment for multiple comparisons was made. Two samples were excluded during analysis due to poor sample quality (defective acquisition and sample collection). Correlations between numerical values were evaluated by Spearman and Pearson rank correlation tests.

## Results

### Clinical and demographic characteristics of study participants

The study cohort comprised 28 patients with AQP4 + NMOSD, 19 patients with MOGAD and 45 HC. Characteristics of all the study participants are summarized in Table 1. AQP4 + NMOSD patients had a higher rate of autoimmune comorbidities (7/28) compared to MOGAD (1/19). The majority of AQP4 + NMOSD (22/28) and MOGAD (14/19) patients were treated with relapse preventive immunotherapies at the study visit, mainly rituximab (Table 1).

### Neutrophils from AQP4 + NMOSD patients show an impaired cell death in response to in vitro PMA stimulation

**Fig. 1:**
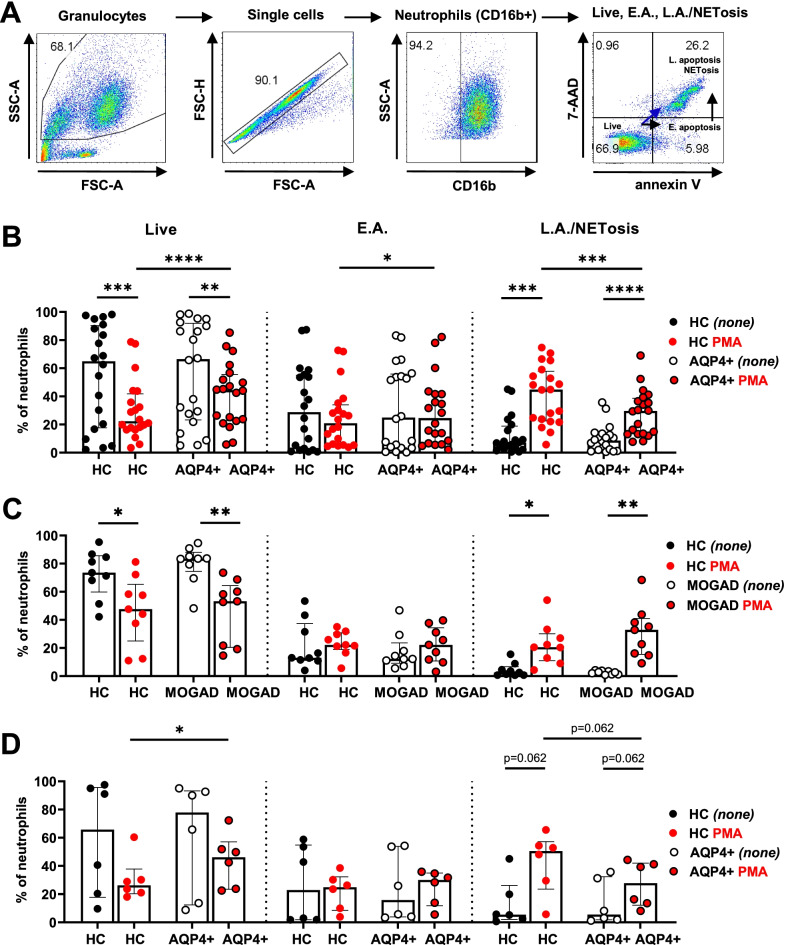
Frequencies of live, early apoptosis and late apoptosis/NETosis neutrophils from patients and HC after PMA exposure. **A** Representative flow cytometry plots and gating strategy to determine the frequencies of living, early apoptosis, late apoptosis/NETotic neutrophils in samples from AQP4 + NMOSD and MOGAD patients and their matched HC. Arrows indicate cell-death trajectory of NETosis and apoptosis pathway. Quantification of the frequencies of spontaneous (black dots, *none*) and PMA-induced cell death (red dots) in **B** AQP4 + NMOSD or **C** MOGAD patients compared to their corresponding HC. **D** AQP4 + NMOSD treatment-naïve patients and HC. Data are represented as median and IQR in scatter plot with a bar graph. Each dot represents an individual subject. *none (unstimulated-vehicle; none).* HC: *n* = 29; AQP4 + NMOSD: *n* = 20; MOGAD: *n* = 9

We first evaluated the capability of neutrophils from AQP4 + NMOSD and MOGAD patients and their corresponding HC to undergo cell death in a PMA-based in vitro assay. Frequencies of living neutrophils or neutrophils undergoing cell death, either spontaneously (*none*) or PMA-induced, were evaluated by annexin V and 7-AAD staining (Fig. 1A). Quantification of spontaneous cell death in unstimulated neutrophils (black dots, *none*) indicated that the proportions of live, early apoptotic (EA) or dying cells (late apoptotic/NETotic cells) were similar in both AQP4 + NMOSD and MOGAD-derived neutrophils (Fig. 1B, C, respectively) when compared with the corresponding unstimulated HC *(none)*.

However, in response to PMA (Fig. 1B, red dots), neutrophils from AQP4 + NMOSD patients showed a higher survival rate (Fig. 1B, live; *p* ˂ 0.0001) and a reduced frequency of dying neutrophils (annexin V^+^7-AAD^+^) compared to their matched HC (Fig. 1B; L.A./NETosis; *p* = 0.0005); meanwhile, no differences were observed between MOGAD patients and their HC (Fig. 1C; L.A./NETosis; *p* = 0.359). We also observed that the frequency of neutrophils at stage of early apoptosis after PMA stimulation was mildly increased in AQP4 + NMOSD patients (Fig. 1B; E.A.; *p* = 0.048), but not in MOGAD patients (Fig. 1C; *p* = 0.669). Thus, although part of the AQP4 + NMOSD neutrophils enter the cell death pathway, they appear to be resistant to completing this process.

Neutrophils from treatment naïve AQP4 + NMOSD patients also showed a trend towards impaired cell death in response to PMA compared to their corresponding HC (Fig. 1D; Live; *p* = 0.031; L.A./NETosis; *n* = *6*; *p* = 0.062), possibly excluding a potential effect of therapies on the observed impaired response of neutrophils to PMA.

### AQP4 + NMOSD-derived neutrophils undergo both apoptosis and NETosis in presence of PMA stimuli

**Fig. 2:**
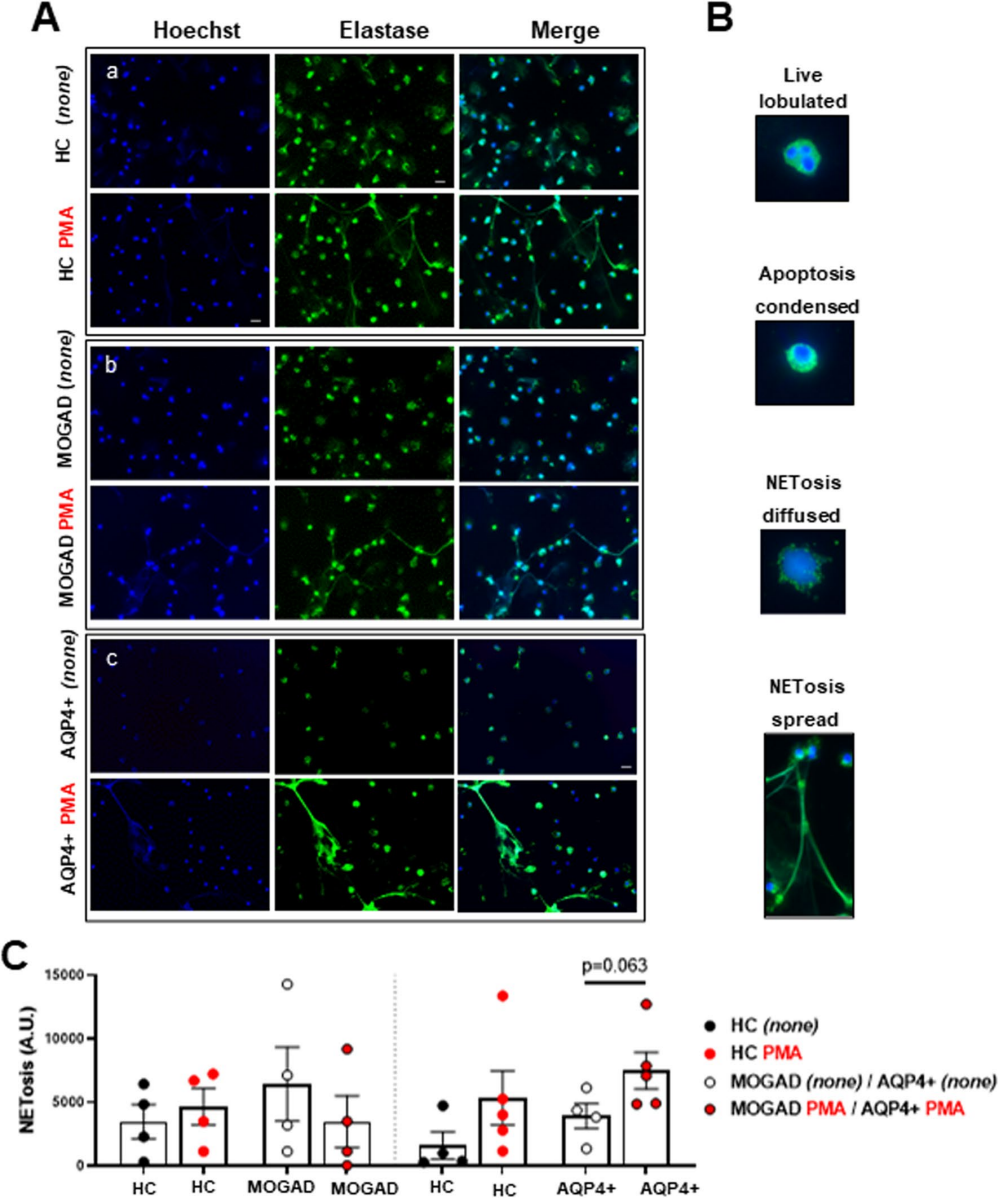
Images of neutrophils’ response to PMA in HC as well as MOGAD and AQP4 + NMOSD patients. **A **Living, apoptotic and NETotic neutrophils from patients and HC. DNA (Hoechst 33342) in blue and neutrophil elastase (NE) in green, as well as merged images, are shown for **a** HC, **b** MOGAD and **c** AQP4 + NMOSD, without PMA (top panels in each group; *none*) and with PMA (lower panels in each group). **B** Neutrophils were assigned into three categories based on their nuclei morphology: live, apoptosis or NETosis. **C** Quantification of NETosis in unstimulated *none* (black dots) and PMA-stimulated neutrophlis (red dots) from MOGAD (left panel) and AQP4 + NMOSD (right panel) patients compared to their corresponding HC. Data are represented as mean ± SEM in scatter plot with a bar graph. Inverted fluorescent microscope at 40 × magnification; scale bar 20 µm. *none (unstimulated-vehicle).* HC: *n* = *9*; AQP4 + NMOSD: *n* = *5*; MOGAD: *n* = *4*

To determine which cell-death pathway is induced by PMA and, thus, impaired in our setup, we evaluated nuclear morphology (DNA staining with Hoechst 33342), neutrophil elastase (NE) distribution and DNA fragments (TUNEL) in unstimulated (*none*) and PMA-stimulated neutrophils from HC, MOGAD and AQP4 + NMOSD patients using fluorescence microscopy (Fig. 2).

Figure 2A, B and Additional file 1: Figure S1 show that PMA appears to induce early apoptosis (condensed (pyknotic) nuclei), NETosis (delobulated or spread nuclei co-localized with elastase), as well as late apoptosis (TUNEL-positive cells) in isolated neutrophils. PMA-induced NETosis was observed in neutrophils from AQP4 + NMOSD patients (Fig. 2C; right panel; p = 0.063). No effect of PMA was observed in HC and MOGAD neutrophils (Fig. 2C).

### Modulation of caspase-3 activation in response to PMA differentiates AQP4 + NMOSD from HC neutrophils

**Fig. 3:**
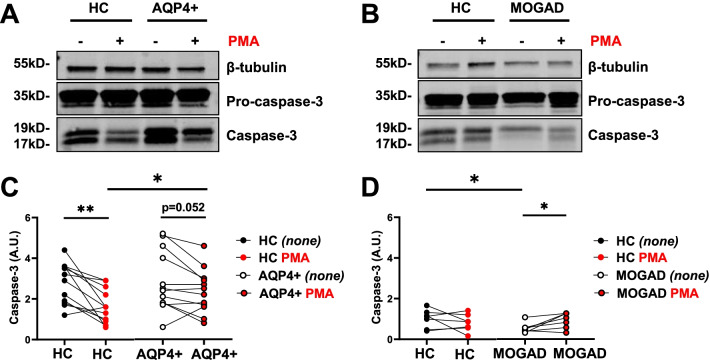
Caspase-3 expression in neutrophils from AQP4 + NMOSD and MOGAD patients and HC. Neutrophils from patients and age- and sex- matched HC were purified and processed for western blot simultaneously. Cell lysates were obtained after incubation with and without PMA. Representative western blot images of **A** AQP4 + NMOSD and **B** MOGAD neutrophil lysates compared to their corresponding HC. **C**, **D** Levels of caspase-3 (19 kDa and 17 kDa) normalized to β-tubulin shown as caspase-3 (A.U.) in unstimulated (black dots; *none*) and PMA-stimulated neutrophils (red dots). Each dot represents the mean value of 2 or 3 independent WB for each patient and corresponding HC. Data are represented with lines connecting among unstimulated and PMA-stimulated samples. *none (unstimulated-vehicle).* HC: *n* = *19*; AQP4 + NMOSD: *n* = *12*; MOGAD: *n* = *7*

Our data so far indicate an impaired neutrophil cell death in response to PMA in AQP4 + NMOSD patients and confirmed that the annexin V^+^ 7-AAD^+^ cell fraction includes cells in advanced stage of apoptosis, but also cells undergoing NETosis. Therefore, we next investigated which cell-death pathway is deficient. To assess whether the apoptotic or NETotic response is deficient in AQP4 + NMOSD-derived neutrophils, levels of active caspase-3 upon PMA stimulation in AQP4 + NMOSD, MOGAD and HC neutrophils were investigated (Fig. 3). While HC neutrophils showed a pronounced PMA-induced reduction of caspase-3 activation (Fig. 3C; *p* = 0.002), this effect was moderate in AQP4 + NMOSD-derived neutrophils (*p* = 0.052). No difference in caspase-3 activation was found in unstimulated samples (*none*) from AQP4 + NMOSD patients compared to their matched HC (Fig. 3C; black dots, *p* = 0.785). In response to PMA, levels of caspase-3 were elevated in AQP4 + NMOSD-derived neutrophils compared to HC (Fig. 3C; red dots; p = 0.016). Effects of PMA on caspase-3 was not observed in MOGAD and corresponding HC (Fig. 3D; p = 0.578). No difference was found in caspase-3 activation in neutrophils from MOGAD patients compared to their HC counterpart after PMA stimulation (Fig. 3D; red dots, *p* = 0.375). A mildly decreased caspase-3 activation was observed in MOGAD without PMA in comparison to their matched HC (Fig. 3D; black dots, p = 0.031).

Thus, the observed abnormal response to PMA in AQP4 + NMOSD neutrophils was also supported by this deficient reduction of caspase-3 activation in response to PMA.

### Soluble markers of NETosis are not affected in cultured AQP4 + NMOSD neutrophils

**Fig. 4:**
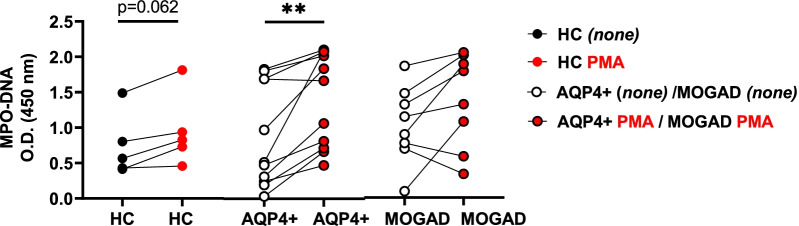
Spontaneous and PMA-induced generation of MPO-DNA by AQP4 + NMOSD and MOGAD neutrophils. MPO-DNA complexes were analyzed by ELISA in neutrophil culture supernatants from AQP4 + NMOSD and MOGAD patients and HC. Unstimulated (black dots; *none*) and PMA-stimulated samples (red dots) are shown. Each dot represents an individual subject. Lines connect the unstimulated and PMA-stimulated data points of the same patient. *None (unstimulated-vehicle).* HC: *n* = *5*; AQP4 + NMOSD: *n* = *11*; MOGAD: *n* = *8*

We next determined if NETosis may be affected in AQP4 + NMOSD neutrophils by analyzing levels of MPO-DNA in culture supernatants of HC, AQP4 + NMOSD and MOGAD neutrophils with and without (*none*) PMA stimulation. As indicated in Fig. 4, after PMA exposure, MPO-DNA levels were slightly increased in HC (*p* = 0.062), and significantly elevated in AQP4 + NMOSD compared to their unstimulated counterparts (*p* = 0.002). However, PMA did not lead to an increase in MPO-DNA in cultured neutrophils from MOGAD patients compared to the unstimulated HC. No differences were found in spontaneous and PMA-induced MPO-DNA levels when comparing HC, AQP4 + NMOSD, and MOGAD groups.

### ROS generation is not affected in AQP4 + NMOSD and MOGAD-derived granulocytes after PMA stimulation

As depicted in Figure S2 (Additional file 2; left panel), granulocytes from both AQP4 + NMOSD patients and HC produced ROS in response to PMA stimulation (*p* = 0.004 and *p* = 0.004, respectively). In AQP4 + NMOSD, a mild increase in PMA-induced ROS production compared to their HC counterpart was observed (see Figure S2A, Additional file 2; red dots, *p* = 0.039). In MOGAD, an increase in ROS production was also found in response to PMA stimulation (*p* = 0.008). However, no differences were found in PMA-induced ROS production in MOGAD patients compared to their matched HC (see Figure S2A; Additional file 2; right panel, *p* = 0.250). Thus, ROS generation in response to PMA seems to be unaltered in granulocytes from AQP4 + NMOSD and MOGAD patients compared to their unstimulated samples. However, in MOGAD patients, a mild decrease in ROS production was observed in unstimulated samples compared to their unstimulated HC counterpart (see Figure S2B; Additional file 2; *p* = 0.016).

### cfDNA is elevated in serum samples of AQP4 + NMOSD and is associated with serum NfL levels

**Fig. 5:**
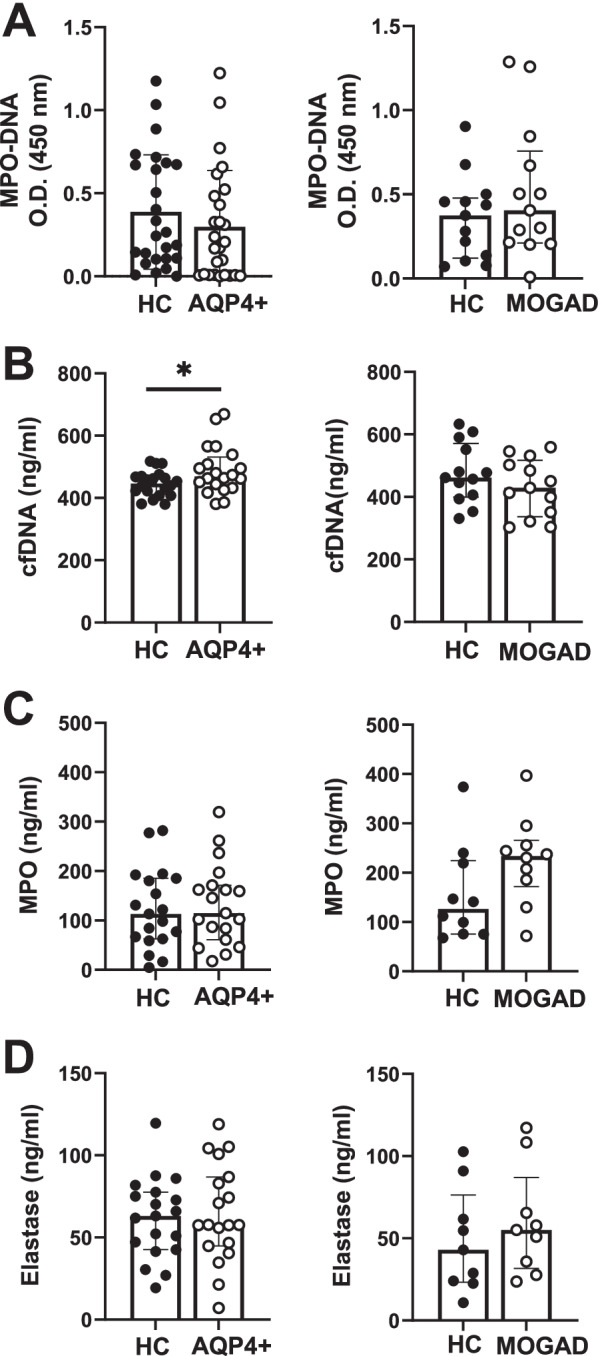
MPO-DNA, cfDNA, MPO, and elastase in serum samples from AQP4 + NMOSD and MOGAD patients and HC. Serum samples from AQP4 + NMOSD (left panels) and MOGAD patients (right panels) and their corresponding age- and sex- matched HC were analyzed by ELISA for MPO-DNA (**A**), cfDNA (**B**), MPO (**C**) and elastase (**D**). Data are represented as median and IQR in scatter plot with a bar graph. Each dot represents an individual subject. AQP4 + NMOSD: *n* = *26(A), n* = *20(B), n* = *19(C), n* = *19(D); MOGAD: n* = *13(A), n* = *13(B), n* = *10(C), n* = *9(D) and corresponding matched HC.* The patient cohorts included in the data were not identical for each investigated analyte

Next, we investigated circulating neutrophil-associated markers such as MPO-DNA, cell-free DNA (cfDNA), MPO and elastase in serum samples of AQP4 + NMOSD and MOGAD patients as well as their corresponding HC (Fig. 5).

The analysis of NETosis-associated markers revealed a mild increase of cfDNA in AQP4 + NMOSD but not in MOGAD serum samples compared to their HC (Fig. 5B, *p* = 0.040). However, no difference was found in serum levels of MPO-DNA (Fig. 5A; *p* = 0.315 and *p* = 0.376), MPO (Fig. 5C; *p* = 0.738 and *p* = 0.105) or neutrophil elastase (Fig. 5D; *p* = 0.829 and *p* = 0.30) in AQP4 + NMOSD and MOGAD compared to their HC.

**Fig. 6:**
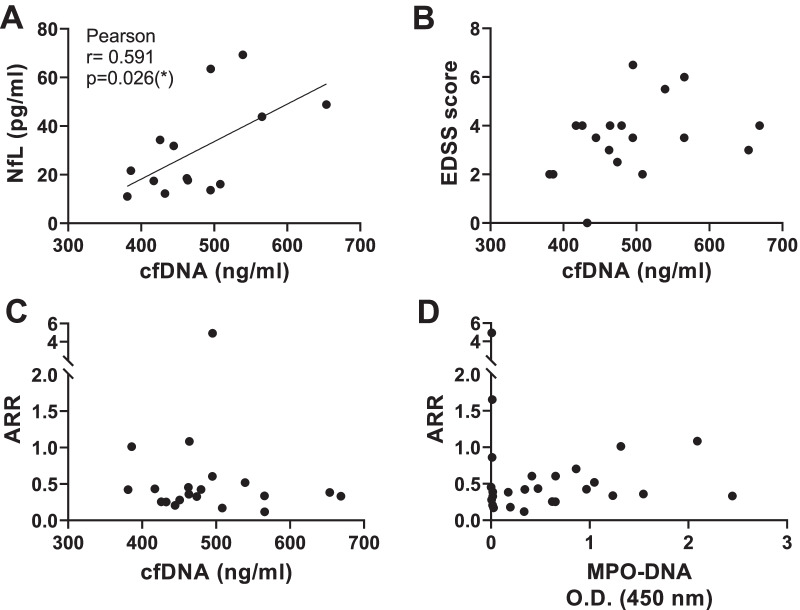
Correlations between serological markers of neutrophil death with NfL and the clinical parameters EDSS and ARR. **A** Pearson correlation among cfDNA and NfL. **B** Spearman correlation between cfDNA and EDSS. **C** Pearson correlation among cfDNA and ARR. **D** Pearson correlation among MPO-DNA and ARR. Each dot represents an individual subject. AQP4 + NMOSD: *n* = *14(A), n* = *19(B), n* = *20(C), n* = *26(D).* The patient cohort included in the correlation cfDNA / ARR was not identical to the cohort analyzed in the correlation MPO-DNA/ARR

Next, we aimed to evaluate potential associations between neutrophil-death biomarkers cfDNA (Fig. 5B) and EDSS, cfDNA and MPO-DNA with annualized relapse rate (ARR) and NfL. Serum levels of cfDNA were positively correlated with levels of NfL in AQP4 + NMOSD patients (Fig. 6A; *r* = 0.591, *p* = 0.026). However, no association was found among cfDNA and EDSS or ARR and MPO-DNA and ARR in AQP4 + NMOSD (Fig. 6; *p* = 0.083; *p* = 0.865 and *p* = 0.578, respectively). In MOGAD patients, no correlations were found between serum cfDNA and NfL, EDSS or ARR (data not shown).

### Increased levels of anti-apoptotic cytokines in AQP4 + NMOSD patients’ serum

We next evaluated anti-apoptotic mediators in serum, which might contribute to the resistance of AQP4 + NMOSD neutrophils to undergo apoptosis.

Serum levels of GM-CSF, IL-6, IL-8, IL-15, TNF-ɑ and IL-10 were assessed as described in the *Methods* section. While IL-15 was increased in both AQP4 + NMOSD and MOGAD patients compared to their matched HC (see Figure S3, Additional file 3, *p* = 0.003 and *p* = 0.003, respectively), IL-10 and GM-CSF were only elevated in AQP4 + NMOSD patients (see Figure S3, Additional file 3, *p* = 0.0001; *p* = 0.013, respectively).

## Discussion

In our previous study, we showed that NMOSD neutrophils are functionally impaired [25]. Now, we investigated if alterations in cell-death pathways could be an additional impairment affecting NMOSD neutrophils. An increased cell survival may lead to neutrophil accumulation, observed in CSF and active CNS lesions of AQP4 + NMOSD and MOGAD patients [13, 14, 39].

We used PMA-based in vitro assays to investigate neutrophil cell death. PMA has been extensively used to study NADPH-oxidase 2 (NOX2)-ROS-dependent NETosis in vitro [28, 40–43], but also to study apoptosis and NETosis together [44]. Previous studies reported low or no apoptosis in response to PMA in human healthy neutrophils [45, 46]. Our data indicated however, that PMA-induced translocation of phosphatidylserine to membranes and consequent annexin V binding, a hallmark of early phases of apoptosis, as well as apoptotic DNA fragmentation as demonstrated by TUNEL staining took place in our setup. Thus, our results suggest the presence of both types of cell death in neutrophil cultures exposed to PMA (Figs. 1, 2, 3, and Additional file 1: Figure S1) as previously reported [44].

Using this setting, we observed that AQP4 + NMOSD neutrophils but not MOGAD neutrophils showed an increased neutrophil survival and diminished cell death in response to PMA (Fig. 1B). Thus, it appeared that AQP4 + NMOSD neutrophils were retained in a stage of early apoptosis determined by annexin V staining (Fig. 1B). It is unlikely that this observation was a mere treatment effect since the same trend was observed in AQP4 + NMOSD treatment-naïve patients (Fig. 1D; *n* = *6*). It has been reported that PMA-induced NETosis involves apoptosis inhibition [47] and inhibition of caspase-3 activation in healthy neutrophils. Despite the differences in early apoptosis and the presence of DNA fragmentation observed in our study, our data confirmed those results and showed a significant reduction of caspase-3 activation after PMA exposure, mainly in HC (*p* = 0.002); while the effect on AQP4 + NMOSD neutrophils was less pronounced (*p* = 0.052). We cannot exclude the possibility that after a shorter stimulation with PMA, the effects on caspase-3 may differ from those observed after 2.5 h of stimulation. Further studies on the regulation of caspase-3 over time, in response to PMA, are needed to understand whether apoptosis and NETosis are coexisting in our cell culture, or even at single cell levels as previously reported by Azzouz et al. [45], or whether these types of cell death occur with different kinetics in AQP4 + NMOSD neutrophils.

Neutrophils have the capacity to switch between apoptosis to NETosis and vice versa [42, 44, 47, 48], probably as compensatory mechanisms [42]. The inhibition of caspase-3 activation by PMA may induce a switch to NETosis, as previously demonstrated [47]. To evaluate this, we investigated in a subgroup of patients if NETosis was the form of cell death impaired in PMA-stimulated AQP4 + NMOSD neutrophils. We showed, however, that (NOX2)-ROS-dependent NETosis in response to PMA was not affected, and a trend towards an increase in NETosis was found in neutrophils from AQP4 + NMOSD compared to their unstimulated counterpart (Fig. 2; *p* = 0.063). In line with these results, we further demonstrated that AQP4 + NMOSD neutrophils generated higher levels of MPO-DNA after PMA exposure (Fig. 4; *p* = 0.002). PMA-induced MPO-DNA in MOGAD and HC was not significant, possibly due to the heterogeneous response observed within the MOGAD group (Fig. 4; *p* = 0.109) and the limited sample size in HC group (Fig. 4*,* HC; *p* = 0.062, *n* = *5*). Thus, related to the production of MPO-DNA, no major differences were observed between neutrophils from patients and HC. In agreement with this, serum levels of MPO-DNA, MPO and elastase did not differ between AQP4 + NMOSD, MOGAD and their matched HC, respectively (Fig. 5).

Thus, in response to PMA, AQP4 + NMOSD neutrophils showed higher survival rates, seemed to be partly retained in the phase of early apoptosis, showed diminished inhibition of caspase-3 activation, but were able to undergo NETosis. A prolonged neutrophil survival or delay in neutrophil apoptosis has been reported in several inflammatory diseases [49] such as ANCA-AAV [50], rheumatoid arthritis (RA) [29, 50], cystic fibrosis [48], inflammatory bowel disease [51], lung cancer [52], sepsis [53] and acute pancreatitis [54]. In contrast to these reports that evaluated spontaneous apoptosis at later timepoints (24 h) [48, 50, 52], we observed, at an earlier time point of culture, an impaired cell death only in neutrophils stimulated with PMA. Thus, it remains to be elucidated whether AQP4 + NMOSD neutrophils would also show compromised spontaneous cell death at later time points of culture.

Additionally, in serum samples from AQP4 + NMOSD but not from MOGAD patients, a mild increase of cfDNA was observed. CfDNA can be released during neutrophil apoptosis and NETosis [55], and increased cfDNA levels have been associated with disease activity, in patients with SLE, RA [55] and, very recently, also in patients with AQP4 + NMOSD [27]. Interestingly, in this latter study, Murata et al. demonstrated that serum cfDNA in AQP4 + NMOSD was derived mainly from neutrophils [27]. Thus, it is plausible that the increased serum cfDNA found in AQP4 + NMOSD is of neutrophil origin (Fig. 5B). Increasing evidence supports that NfL, a biomarker of neuronal damage, is as a good biomarker of disease activity and disability in AQP4 + NMOSD [37, 38]. Remarkably, we found a positive correlation among serum cfDNA and NfL in AQP4 + NMOSD patients (Fig. 6A), suggesting that both biomarkers could be combined to better assess disease progression in AQP4 + NMOSD. However, in agreement with Murata et al. [27], no associations were observed among cfDNA and EDSS or ARR (Fig. 6B, C; *p* = 0.083 and  = 0.865, respectively). Unfortunately, due to sample limitations and the incapacity to analyze cfDNA, ROS and MPO-DNA in the same patients, associations between cfDNA levels and the values of ROS or MPO-DNA obtained from the in vitro assays, could not be assessed. Such an analysis could have further underlined the potential translational relevance of our findings.

Neutrophil apoptosis can be modulated by internal signals (ROS) [49] and external signals such as cytokines GM-CSF, IL-6, IL-8, IL-15 and TNF-ɑ [56–58]. Additionally, IL-10 has been associated with an increase in apoptosis rates in vivo [56]. Consistent with what was previously published [26], we found no reduction in ROS production in AQP4 + NMOSD neutrophils stimulated with PMA (see Figure S2A, Additional file 2), but an increase of the pro-apoptotic IL-10 and the anti-apoptotic GM-CSF in serum from AQP4 + NMOSD patients compared to HC (see Figure S3, Additional file 3, 3A and 3F, respectively). This is in line with previous reports on increased GM-CSF levels in the CSF of AQP4 + NMOSD patients, but not in MOGAD patients, compared to HC [59]. GM-CSF, a potent suppressor of neutrophil apoptosis, which triggers the expression of anti-apoptotic factors like Mcl-1 [53], has been described to act synergistically with granulocyte-colony stimulating factor (G-CSF) to modulate the activity of neutrophils, delaying neutrophil apoptosis and promoting granulopoiesis in the bone marrow [60]. Previous studies in patients and in a mouse model of AQP4 + NMOSD suggest a potential contribution of G-CSF to raising circulating granulocytes counts, CNS lesion severity, and disease exacerbation [18, 22].

On the other hand, an increase in IL-10 was observed in serum samples from AQP4 + NMOSD patients but not in those from MOGAD patients (see Figure S3F; Additional file 3). This is in line with the reported increased IL-10 levels in CSF from AQP4 + NMOSD patients but not in MOGAD patients [61]. IL-10 is considered an anti-inflammatory cytokine, which seems to inhibit the GM-CSF-mediated anti-apoptotic effects [49]. However, in our setup, the survival of activated neutrophils in AQP4 + NMOSD may increase in response to GM-CSF but fails to be down-modulated by IL-10. This is a point that needs to be investigated in future studies.

We also observed a significant increase of IL-15 in the serum of both AQP4 + NMOSD and MOGAD patients compared to HC. This is in line with previous reports that showed elevated IL-15 in serum and CSF samples from NMOSD patients [59, 62]. Interestingly, IL-15 has been reported to enhance neutrophil effector functions and to exacerbate their migration and proinflammatory activities through the delay of neutrophil apoptosis [63]. Apoptosis delay could be promoted directly by the reported IL-15-mediated down-modulation of caspase-3 and caspase-8 activity [64] or, indirectly, by acting on other immune cells and enhancing the expression of neutrophils’ survival factors such as GM-CSF. Thus, elevated levels of both IL-15 and GM-CSF in AQP4 + NMOSD may contribute to the survival of neutrophils in this group of patients.

### Limitations of the study

NMOSD and MOGAD are rare diseases. Therefore, we were limited by the number of available patients. Moreover, most of the patients received immunotherapies that could impact on the study results. To partly overcome this limitation, we collected samples over several years (2017—2021). Additionally, neutrophils are cells extremely sensitive to environmental conditions, and their functionality can be affected by several factors such as age and sex of the donor, or the time of sample collection. Therefore, to allow comparability between groups, we compared in all our analyses patients with their corresponding age- and sex- matched HC, whose samples were collected, processed, and studied simultaneously to patients’ samples, except for data shown in Fig. 4. A direct comparison between AQP4 + NMOSD and MOGAD was consequently not feasible because the two groups of patients were not matched for age and sex, and data acquisition occurred on distinct days. Similarly, the evaluation of a potential association of cfDNA with ROS or MPO-DNA was not possible since the data on cfDNA were not obtained from exactly the same cohort as the in vitro data for the other analytes. Another limitation of our study is the missing data on neutrophil counts for most of the patients and for all HC. Hemogram analysis were not part of the study protocol for this prospective study at the time of sampling. Therefore, we could not correlate absolute neutrophil counts with the observed increased neutrophil survival. In addition, due to reduced access to patient samples, the use of different cell-death inducers other than PMA was not feasible. This is an evident limitation since PMA is principally a NETosis inducer, and comparisons between different stimuli are missing. Finally, we could not exclude that neutrophil death was influenced by other factors that we did not consider in our analyses, such as concomitant autoimmune diseases, which are frequently present in NMOSD [30], and patients’ treatment status.

## Conclusion

Taken together, our data show a different response to PMA in neutrophils from AQP4 + NMOSD and MOGAD patients when compared to their corresponding HC. Neutrophils from AQP4 + NMOSD patients are characterized by an increased survival after PMA exposure in vitro. An increased lifespan, accompanied by neutrophil accumulation, may contribute to the maintenance of a proinflammatory environment within the CNS. This impaired neutrophil cell death distinguishes AQP4 + NMOSD and MOGAD neutrophils, corroborating the notion that AQP4 + NMOSD and MOGAD are pathogenetically different diseases.

## Supplementary Information

**Additional file 1: Figure S1.** Images of neutrophils response to PMA in HC as well as AQP4 + NMOSD patients. Representative fluorescent microscope image of neutrophils from HC (top panel) and AQP4 + NMOSD (lower panel) after exposure to PMA. DNA (Hoechst 33342) in blue, neutrophil elastase (NE) in green and DNA fragments (TUNEL) as well as merged images are shown. Images obtained at 40 × magnification; scale bar 20 µm. HC: *n* = *3*; AQP4 + NMOSD: *n* = *3.*

**Additional file 2: Figure S2.** Evaluation of spontaneous and PMA induced ROS production in AQP4 + NMOSD, MOGAD patients and HC granulocytes. Granulocytes from patients and matched HC were isolated and analyzed simultaneously and cultured without (black dots, *none*) and with PMA (red dots). Intracellular ROS were indirectly assessed by flow cytometry analysis of DHR 123. Depicted is the mean fluorescence intensity (MFI) of oxidized-DHR123. A) Data are represented with lines connecting the unstimulated and PMA-stimulated data points of the same patient sample B) Depicted are the data from unstimulated and MOGAD samples in a different scale to show group differences. Each dot represents an individual subject. HC: *n* = *17*; AQP4 + NMOSD: *n* = *9*; MOGAD: *n* = *8*.

**Additional file 3: Figure S3.** Evaluation of serum cytokines and chemokines in AQP4 + NMOSD, MOGAD patients and HC. Levels of GM-CSF (A), IL-6 (B), IL-8 (C), IL-15 (D), TNF-alpha (E) and IL-10 (F) were investigated using the Meso Scale Diagnostics MULTISPOT Assay System in serum samples from AQP4 + NMOSD (left panel) and MOGAD (right panel) patients compared with their respective HC. Data are represented as median and IQR in scatter plot with a bar graph. Each dot represents an individual subject. HC: *n* = 37; AQP4 + NMOSD: *n* = *24*; MOGAD: *n* = *13.*

## Data Availability

The datasets generated and/or analyzed during the current study are not publicly available due to local regulations concerning protection of patient data, but on reasonable request, approval for distribution of data will be obtained from the institutional review board of Charité-Universitätsmedizin Berlin and anonymized data will be made available by the corresponding author.

## Abbreviations

AQP4+: Aquaporin-4-immunoglobulin-G-positive (AQP4-IgG)
cfDNA: Cell-free double strand deoxyribonucleic acid
GM-CSF: Granulocyte monocyte-colony stimulating factor
HC: Healthy controls
MOGAD: Myelin oligodendrocyte glycoprotein antibody-associated disease
MPO-DNA: Myeloperoxidase-double strand deoxyribonucleic acid complexes
NETs: Neutrophil extracellular traps
NMOSD: Neuromyelitis optica spectrum disorders
PMA: Phorbol 12-myristate 13-acetate
7-AAD: 7-Amino-actinomycin D
