# Supplementary figures and images for: Impaired response of blood neutrophils to cell-death stimulus differentiates AQP4-IgG-seropositive NMOSD from MOGAD
Source: J Neuroinflammation. 2022 Oct 1;19:239. doi: 10.1186/s12974-022-02600-0 (PMC9526338; doi:10.1186/s12974-022-02600-0)

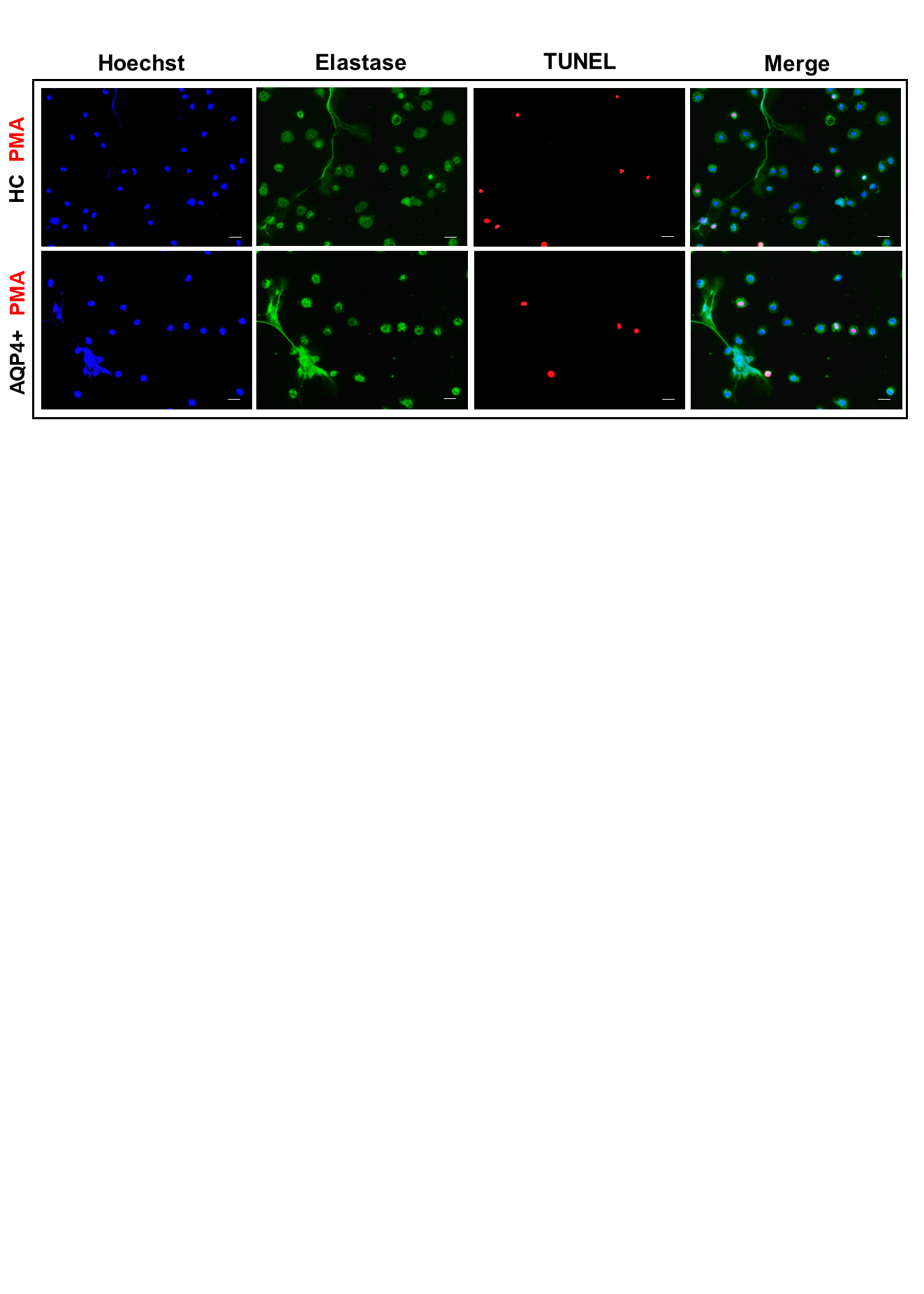

Supplement: Supplementary file 1 — Additional file 1: Figure S1. Images of neutrophils response to PMA in HC as well as AQP4 + NMOSD patients. Representative fluorescent microscope image of neutrophils from HC (top panel) and AQP4 + NMOSD (lower panel) after exposure to PMA. DNA (Hoechst 33342) in blue, neutrophil elastase (NE) in green and DNA fragments (TUNEL) as well as merged images are shown. Images obtained at 40 × magnification; scale bar 20 µm. HC: n = 3; AQP4 + NMOSD: n = 3. [file 12974_2022_2600_MOESM1_ESM.tif]

**A**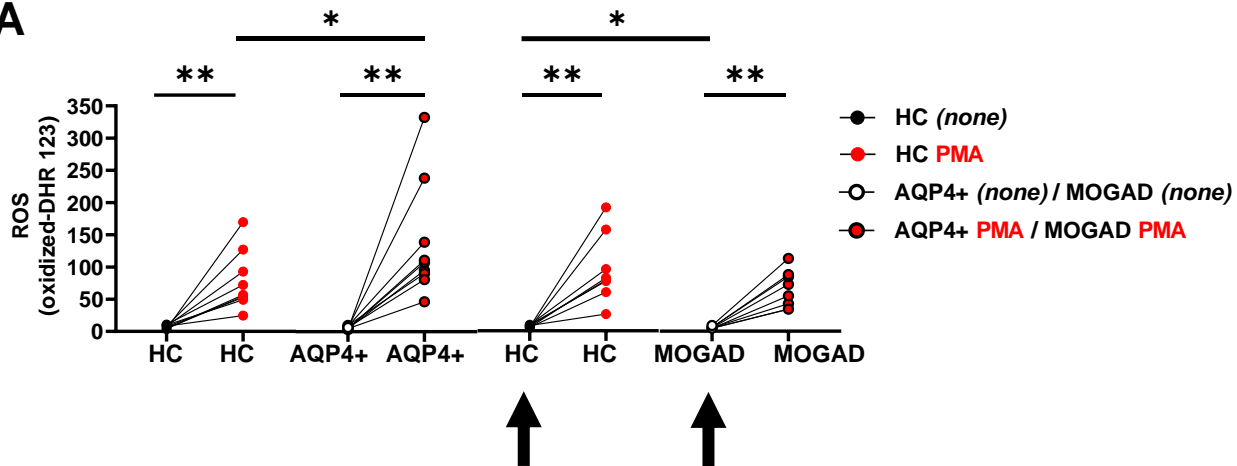**B**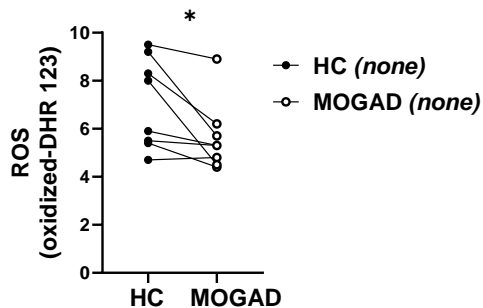

Supplement: Supplementary file 2 — Additional file 2: Figure S2. Evaluation of spontaneous and PMA induced ROS production in AQP4 + NMOSD, MOGAD patients and HC granulocytes. Granulocytes from patients and matched HC were isolated and analyzed simultaneously and cultured without (black dots, none) and with PMA (red dots). Intracellular ROS were indirectly assessed by flow cytometry analysis of DHR 123. Depicted is the mean fluorescence intensity (MFI) of oxidized-DHR123. A) Data are represented with lines connecting the unstimulated and PMA-stimulated data points of the same patient sample B) Depicted are the data from unstimulated and MOGAD samples in a different scale to show group differences. Each dot represents an individual subject. HC: n = 17; AQP4 + NMOSD: n = 9; MOGAD: n = 8. [file 12974_2022_2600_MOESM2_ESM.pdf]
