# Supplementary material for: Impaired response of blood neutrophils to cell-death stimulus differentiates AQP4-IgG-seropositive NMOSD from MOGAD
Source: J Neuroinflammation. 2022 Oct 1;19:239. doi: 10.1186/s12974-022-02600-0 (PMC9526338; doi:10.1186/s12974-022-02600-0)
Supplement: Supplementary file 3 — Additional file 3: Figure S3. Evaluation of serum cytokines and chemokines in AQP4 + NMOSD, MOGAD patients and HC. Levels of GM-CSF (A), IL-6 (B), IL-8 (C), IL-15 (D), TNF-alpha (E) and IL-10 (F) were investigated using the Meso Scale Diagnostics MULTISPOT Assay System in serum samples from AQP4 + NMOSD (left panel) and MOGAD (right panel) patients compared with their respective HC. Data are represented as median and IQR in scatter plot with a bar graph. Each dot represents an individual subject. HC: n = 37; AQP4 + NMOSD: n = 24; MOGAD: n = 13. [file 12974_2022_2600_MOESM3_ESM.pdf]

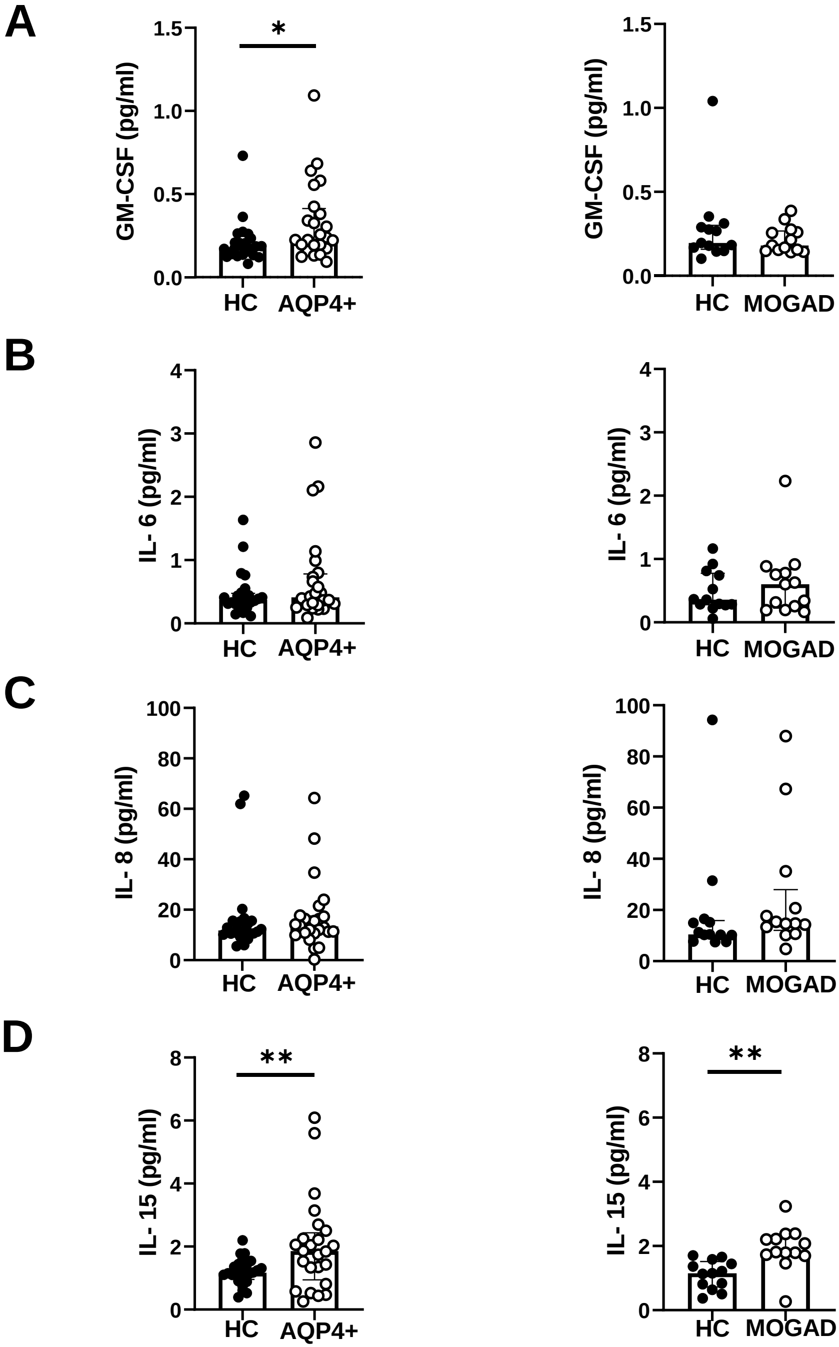
A
B
C
D
GM-CSF (pg/ml)
IL- 6 (pg/ml)
IL- 8 (pg/ml)
IL- 15 (pg/ml)
HC
AQP4+
MOGAD
*
**
**

E

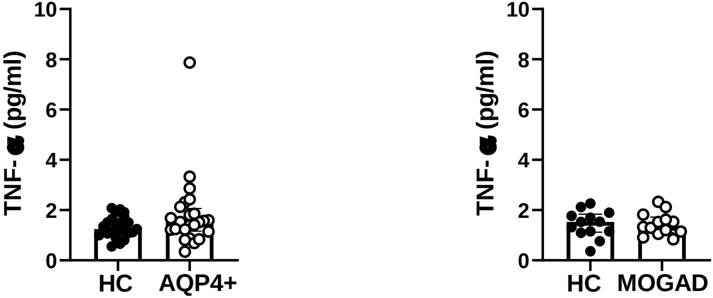
TNF- α (pg/ml)
10
8
6
4
2
0
HC
AQP4+
TNF- α (pg/ml)
10
8
6
4
2
0
HC
MOGAD

F

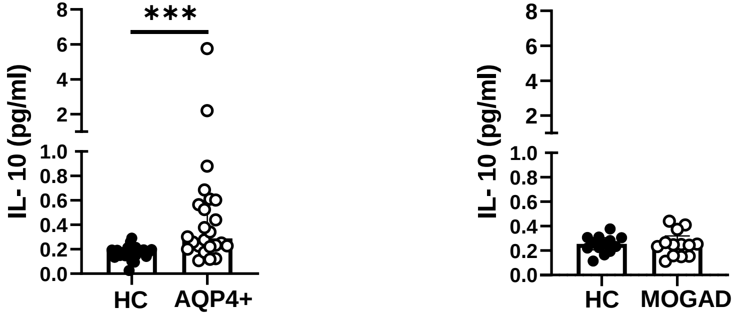
***
IL- 10 (pg/ml)
8
6
4
2
1.0
0.8
0.6
0.4
0.2
0.0
HC
AQP4+
IL- 10 (pg/ml)
8
6
4
2
1.0
0.8
0.6
0.4
0.2
0.0
HC
MOGAD
